# FastPros: screening of reaction knockout strategies for metabolic engineering

**DOI:** 10.1093/bioinformatics/btt672

**Published:** 2013-11-19

**Authors:** Satoshi Ohno, Hiroshi Shimizu, Chikara Furusawa

**Affiliations:** ^1^Department of Bioinformatic Engineering, Graduate School of Information Science and Technology, Osaka University, 1-5 Yamadaoka, Suita, Osaka 565-0871 and ^2^Quantitative Biology Center (QBiC), RIKEN, 6-2-3 Furuedai, Suita, Osaka 565-0874, Japan

## Abstract

**Motivation:** Although constraint-based flux analysis of knockout strains has facilitated the production of desirable metabolites in microbes, current screening methods have placed a limitation on the number knockouts that can be simultaneously analyzed.

**Results:** Here, we propose a novel screening method named FastPros. In this method, the potential of a given reaction knockout for production of a specific metabolite is evaluated by shadow pricing of the constraint in the flux balance analysis, which generates a screening score to obtain candidate knockout sets. To evaluate the performance of FastPros, we screened knockout sets to produce each metabolite in the entire *Escherichia coli* metabolic network. We found that 75% of these metabolites could be produced under biomass maximization conditions by adding up to 25 reaction knockouts. Furthermore, we demonstrated that using FastPros in tandem with another screening method, OptKnock, could further improve target metabolite productivity.

**Availability and implementation:** Source code is freely available at http://www-shimizu.ist.osaka-u.ac.jp/shimizu_lab/FastPros/, implemented in MATLAB and COBRA toolbox.

**Contact:** chikara.furusawa@riken.jp or shimizu@ist.osaka-u.ac.jp

**Supplementary information:** Supplementary data are available at *Bioinformatics* online.

## 1 INTRODUCTION

Metabolic engineering of microbes has been successfully used for the production of a variety of useful compounds in microbes (Keasling, 2010; Shimizu, 2002; Stephanopoulos *et al.*, 1998; Zaldivar *et al.*, 2001). Moreover, genetic modification techniques have enabled the disruption and ‘rewiring’ of metabolic fluxes to improve the production of target products (Atsumi *et al.*, 2008; Becker *et al.*, 2011). Owing to the complexity of metabolic systems, however, achieving a desired metabolic state through genetic modification remains difficult. Given that several individual genetic modifications are often required to improve target productivity (Becker *et al.*, 2011; Yim *et al.*, 2011), the selection of an appropriate set of modifications from a large number of possible combinations is challenging. To overcome this obstacle, tools based on computer simulation and mathematical modeling have been developed, which make possible screening for appropriate sets of genetic modifications to improve target productivity (Burgard *et al.*, 2003; Patil *et al.*, 2005; Tepper and Shlomi, 2010). Flux balance analysis (FBA) is a widely used method for estimating metabolic fluxes using genome-scale metabolic models (GSMs) (Feist and Palsson, 2008; Oberhardt *et al.*, 2009). In FBA, metabolic fluxes can be quantitatively estimated by assuming a steady state metabolic system and optimization of an objective function. Maximization of biomass production flux has been generally adopted as the objective function, and it has been demonstrated that metabolic fluxes estimated by maximizing biomass production are in agreement with experimentally obtained fluxes (Varma and Palsson, 1994; Yoshikawa *et al.*, 2011). Therefore, quantitative flux predictions by FBA can accelerate the rational design of metabolic networks to improve the yield of target products (Alper, *et al.*, 2005; Park, *et al.* 2007).

*In silico* screening of genetic modifications is a widely used application of FBA for metabolic engineering, and to date several such algorithms have been proposed (Tomar and De, 2013; Zomorrodi *et al.*, 2012). These algorithms can be classified into two categories: comprehensive screening and iterative screening. For example, the popular comprehensive knockout screening algorithm OptKnock, which has been used in both academic and industrial settings (Fong *et al.*, 2005; Pharkya and Maranas, 2006; Yim *et al.*, 2011), identifies the reaction knockouts that hold the most promise for achieving the highest target production yield among all possible sets of reaction knockouts (Burgard *et al.*, 2003). However, as the calculation time increases exponentially as a function of the number of reaction knockouts that are simultaneously present in the original network, the maximum number of reaction knockouts to which this strategy can be applied is limited.

Iterative screening for increasing the target productivity can be carried out at relatively low computational costs. In a simple iterative knockout screening method, the effects of all possible single reaction knockouts on metabolic fluxes are evaluated in the first iteration, and the reactions whose knockouts result in the highest score (e.g. highest production yields under biomass production maximization) are selected. In the second iteration, in addition to the reaction knockouts selected in the first iteration, the effects of additional single reaction knockouts are screened. This approach has a drawback, however, of incompletely searching the knockout combinations. For example, when target production yields under biomass maximization are used as a score for screening, iterative screening fails to identify the combinations of two knockouts that show higher production yields only when they are disrupted simultaneously. To overcome this drawback, several systematic screening methods have been developed, such as OptGene (Patil *et al.*, 2005), OptFlux (Rocha *et al.*, 2010), Genetic Design through Local Search (GDLS) (Lun *et al.*, 2009) and Strength Pareto Evolutionary Algorithm (Link *et al.*, 2008). For example, OptGene uses a genetic algorithm to search the vast solution space to obtain optimal knockout sets. Using these approaches, sets of reaction knockouts that improve the target production have been successfully identified with relatively small computational costs. However, if the target production yields are used for the screening score, it is often difficult to identify which sets of multiple knockouts cause the target production that occurs only when the knockouts are done simultaneously. One possible strategy for this problem is to use a screening score that reflects the potential a knockout has to increase the production yields in concert with other knockouts.

In the present study, we developed a new iterative screening algorithm, *F*ast *a*lgorithm of knockout screening for *t*arget *Pro*duction based on *s*hadow price analysis (FastPros), which uses biomass production maximization to identify sets of metabolic reactions whose simultaneous knockouts result in the production of a target metabolite. In this algorithm, we adopted a novel score for iterative knockout screening based on the change in biomass production flux caused by a slight increase in the target production flux, 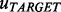. This value corresponds to the shadow price of the constraint of the target-metabolite production flux in the linear programming problem. We demonstrate that 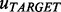 represents the potential of target production and increasing this value by iterative screening of the reaction knockouts generates sets of knockouts that realize the target production. Furthermore, we show that combining FastPros with another tool, OptKnock, provides further improvement of the target production.

## 2 METHODS

### 2.1 Genome-scale metabolic model of *Escherichia coli*

As an original metabolic model, we used a GSM of *E.coli* K-12 MG1655 named iAF1260 (Feist *et al.*, 2007), which contains 1260 open reading frames from the latest genome annotation and over 2000 transport and intracellular reactions. To evaluate the production potential of each cytosolic metabolite in this GSM, we added a transport reaction of the target metabolite if it was absent in the original model, which was assumed to be diffusion transport.

To reduce the computational cost of FBA-based screening, we constructed simplified metabolic models based on our previous study (Ohno *et al.*, 2013), which provides identical flux estimations and screening results to the original model. Briefly, we first identified metabolic reactions whose maximum and minimum fluxes were zero under given environmental conditions by flux variability analysis (Mahadevan and Schilling, 2003) and then removed these reactions from the original model to which target metabolite transporters were added. Second, adjacent metabolic reactions without branching were combined into a single lumped reaction, as the knockout of adjacent reactions results in an identical effect on the flux changes. Third, we identified combined reactions encoded by the same gene sets, as these reactions cannot be separately disrupted in experiments. Finally, knockouts of the reactions encoded by *gapA*, *pgk*, *eno* and *gpmA* were removed from reaction sets, as their reactions appear essential *in vivo* (Baba *et al.*, 2006; Foster *et al.*, 2010). Because the reduced models, including lumped metabolic reactions, were used for the knockout screening, we describe below a knockout using a representative reaction in the lumped reaction.

### 2.2 Flux balance analysis

Constraint-based FBA was performed based on previous studies (Ohno *et al.*, 2013; Orth *et al.*, 2010; Shinfuku *et al.*, 2009). Briefly, a pseudo-steady state of the metabolic profile was assumed, i.e. the net sum of all production and consumption metabolic fluxes for each internal metabolite was set to zero. This assumption resulted in a feasible space that was a convex set in the N-dimensional space of metabolic fluxes (where N stands for the total number of fluxes). In FBA, a particular objective function written as a linear combination of fluxes can be used to calculate the optimal solution at one corner in the feasible flux space. In this study, we used the maximization of biomass production flux as the objective function. After obtaining the maximal biomass production flux by linear programming, we further maximized the production flux of target metabolites under fixed biomass production flux on the maximal value to avoid alternative production flux.

For all simulations, glucose was used as the sole carbon source, and its uptake rate was set to 10 mmol/gDW/h. The oxygen uptake rate was set to 5 mmol/gDW/h, which corresponds to a microaerobic condition, i.e. the oxygen uptake is insufficient to oxidize all NADH produced in glycolysis and the tricarboxylic acid cycle in the electron transfer system. This relatively low oxygen uptake rate was chosen, as higher production yields of target metabolites can be obtained under these conditions, in comparison with the higher oxygen uptake rate when carbon is mainly used to generate biomass and CO_2_. Other external metabolites such as CO_2_ and NH_3_ were allowed to be freely transported through the cell membrane in accordance with a previous study (Feist *et al.*, 2007). All calculations, including linear programming problems, were run using GNU Linear Programming Kit (GLPK) (www.gnu.org/software/glpk/) and MATLAB on a Windows machine with Intel Xeon 2.66 GHz processors. 

### 2.3 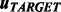: a novel score for knockout screening

Consider the case in which the production flux of a target metabolite is zero under biomass flux maximization. In this case, the increase in the target production flux from zero flux brings about a decrease in the biomass production flux. In contrast, if the target is produced under biomass production maximization, it corresponds to the case that an increase in the target production flux increases the biomass production flux. The change in biomass production flux caused by the increase in target production flux is a useful measure to represent the potential of the target production. In this study, we defined the potential of the target production, 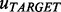, as follows:

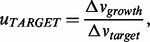

where 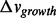 is the change in biomass production flux caused by the increase in the target production flux by 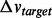 from zero flux. When 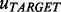 is positive, the target is produced under the biomass production maximization, whereas if it is negative, the target is not produced. Here, the absolute value of 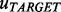 represents the difficulty of altering the sign of this value. When 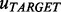 is a small negative value close to zero, a change in its sign can be effected relatively easily by the addition of a single reaction knockout. In contrast, when the value is large and negative, the probability of finding a reaction knockout that changes the sign of 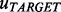 is small. The essence of FastPros is to use 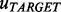 as a score for iterative knockout screening. Even if 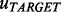 of the wild-type metabolic network is a large negative value, the iterative screening of single reaction knockouts that increase this value can result in approaching a positive 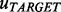, which corresponds to target production under biomass production maximization.


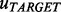
 corresponds to the shadow price of the constraint in which a target production rate is subjected in an FBA problem. The shadow price in a linear programming problem is defined as the small change in the objective function associated with the strengthening or relaxing of a particular constraint. Accordingly, 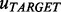 can be calculated using the following linear programming problem:

**Table:** 

max	v_growth_	
( 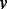 )		
s. t.	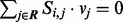	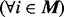
	*v_glc___uptake_* 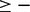 GUR	
	*v_o__2___uptake_* 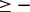 OUR	
	*v_atp___main_* 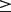 NGAM	
	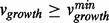	
	*v_j_* = 0	(if reaction *j* is knocked out)
	*v_target_* = *b_target_*	
	*v_j_* 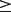 0	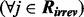
	*v_j_* 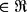	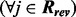

where ***M*** and ***R*** are the set of metabolites and reactions, respectively. *v_j_* is the metabolic flux of reaction *j*. *v_growth_*, *v_glc___uptake_*, *v_o__2___uptake_* and *v_atp___main_* are the biomass production rate, the glucose uptake rate, the oxygen uptake rate and non–growth-associated ATP maintenance requirement, respectively. *S_i,j_* is the stoichiometric coefficient of metabolite *i* in reaction *j*. ***R****_irrev_* and ***R****_rev_* are the set of irreversible and reversible reactions in the metabolic model, respectively, and ***R****_rev_* involves the exchange reactions of available nutrients for a cell, based on a previous article (Feist *et al.*, 2007). *b_target_* was set to 10^−^^5^ to avoid an alternative solution of 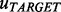. GUR and OUR are the maximum uptake rate of glucose and oxygen, respectively, and were set to 10 and 5 mmol/gDW/h, as described earlier in the text. NGAM (the non–growth-associated ATP maintenance requirement) was set to 8.39 mmol/gDW/h per a previous study (Feist *et al.*, 2007). 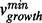, the minimum cell growth rate, was set to 0.05/h, as strains with such a growth rate are anticipated to be difficult to construct experimentally. In this linear programming problem, 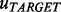 was calculated as the shadow price of the constraint of the target production flux (*v_target_* = *b_target_*).

### 2.4 Screening procedure of FastPros

The screening procedure is schematically illustrated in Supplementary Figure S1. Starting from a reduced metabolic model of *E.coli* with N possible single knockouts, the 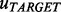 of those networks with all possible double reaction knockouts were calculated. Then, top P knockout sets with regard to this score were chosen for the parent knockout sets. For each generation, all possible single reaction knockouts were further added to the parent sets (leading *P × *N knockout sets), and among these knockout sets that increased 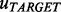 from the parent sets, P knockout sets with the largest 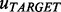 were selected as the parent sets of the next generation. If 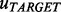 of a selected knockout set became positive or zero, this knockout set was excluded from the iterative screening and was stored as a candidate knockout set for further analysis. The cycle of mutation (additional single reaction knockout) and selection was continued until the number of iterations (i.e. the number of knockouts) reached a maximum number to obtain various sets of reaction knockouts whose additions to the wild-type network result in positive 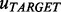 values. Throughout the article, the maximum iteration number was set to 25.

The number of parent knockout sets, P, was determined heuristically. We performed FastPros screening to obtain metabolites whose production is predicted by adding up to 25 reaction knockouts using *P* = 0.5N, *P* = N and *P* = 1.5N. We found that the number of screened metabolites in the case of *P* = 0.5N was significantly smaller than the case of *P* = N (∼18% metabolites screened in *P* = N were failed to be identified), whereas the number of screened metabolites was the same for *P* = N and *P* = 1.5N. The average calculation times for performing the knockout screening of one metabolite by single Xeon CPU (2.66 GHz) were 2.6, 6.2 and 11.4 h for *P* = 0.5N, *P* = N and *P* = 1.5N, respectively. Based on these analyses, we determined *P* = N is appropriate for FastPros screening using the reduced metabolic network of *E.coli* in this study.

## 3 RESULTS

### 3.1 Screening of knockout sets for target production

**Fig. 1.**
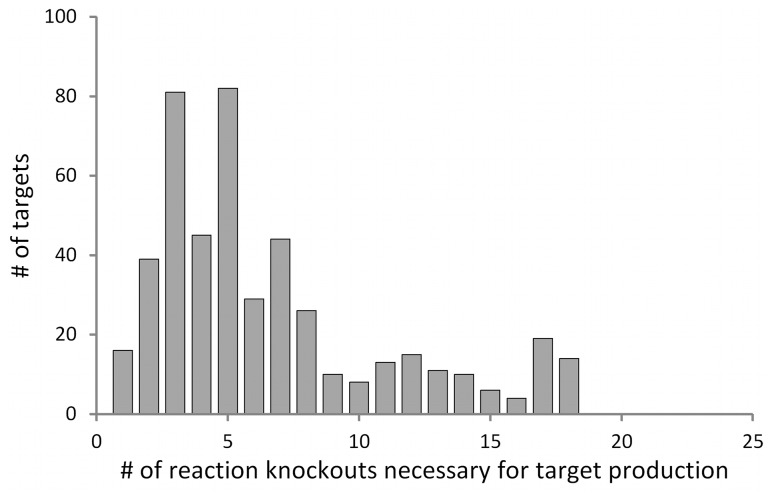
Distribution of the minimum number of reaction knockouts necessary for target metabolite production

To investigate the performance of FastPros, we selected 625 metabolites in the *E.coli* metabolic model and the screened reaction knockout sets that result in the production of each metabolite when the biomass production is maximized. For each target metabolite, iterative screening of the reaction knockouts for increasing the production was performed as described earlier in the text. From this screening, positive or zero 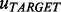 values were obtained for 472 (75%) of the 625 metabolites, including amino acids, nucleic acids, lipids and cofactors by adding up to 25 reaction knockouts to the wild-type metabolic network with each target transporter. This result indicates that production of the corresponding metabolites by the biomass production maximization is possible when adding these selected knockout sets. Figure 1 presents the distribution of the minimum number of reaction knockouts necessary for the target metabolite production. The list of producible metabolites and knockouts necessary for their productions is presented in Supplementary Table S1 (available at http://www-shimizu.ist.osaka-u.ac.jp/shimizu_lab/FastPros/).

The accuracy of the iterative screening by FastPros was evaluated by comparing the screening results to those obtained by the comprehensive screening method OptKnock. Owing to the computational cost, the maximum number of reaction knockouts was set to 3 for OptKnock screening. Comprehensive screening by OptKnock showed that 152 of 625 metabolites are produced when maximizing the biomass production flux by adding three or fewer reaction knockouts. Of the 152 metabolites screened by OptKnock, 136 metabolites were also identified under a maximum of 3 knockouts using our iterative screening based on 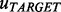, indicating high accuracy of FastPros screening. The other 16 metabolites were also identified by >3 reaction knockouts in FastPros (Supplementary Table S1). It should be stressed that the computational time of comprehensive screening generally increases exponentially with the number of knockouts, and that the maximum number of knockouts is accordingly limited. In contrast, FastPros enables the screening of a larger number of knockouts for target production with low computational cost and has an accuracy that is comparable with that of comprehensive screening methods. In fact, our method demonstrated that a significant number of metabolites exist whose production requires >10 reaction knockouts, an outcome that would be difficult to identify by comprehensive screenings.

Furthermore, we compared the screening performance of FastPros with OptGene and GDLS, which are widely-used iterative knockout screening methods. In the OptGene and GDLS screenings, we adopted the same parameter sets used in the original reports (Lun *et al.*, 2009; Patil *et al.*, 2005) except for the maximum numbers of knockouts. As a result, 247 and 55 metabolites were identified when adding a maximum of 25 reaction knockouts, respectively. On the other hand, using FastPros, we successfully screened 472 metabolites. The results of these screenings are presented in Supplementary Table S1. Among the 247 metabolites screened by OptGene, 244 were also screened by FastPros, whereas all 55 metabolites screened by GDLS were screened by FastPros. As shown in Supplementary Figure S2, although OptGene and GDLS successfully identified knockout sets when using a small number of reaction knockouts, they failed when relatively large numbers of simultaneous knockouts were necessary for the metabolite productions. This result is because the target production flux, which was used as the fitness score, needs to be increased in iterative knockout screenings. The target production flux can be used for the fitness score only when the target metabolites are already produced by the wild-type network or after a small number of simultaneous knockouts. Otherwise, searching the huge number of possible knockout sets without target production makes finding a set of knockouts with non-zero target production extremely difficult. For example, in OptGene, the initial population of knockout sets is usually determined randomly. If there is no set of knockouts that result in the target production in the initial population, selecting knockout sets with higher target production is effectively impossible. In contrast, in FastPros, the use of 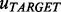 as the fitness score enables us to find reaction knockouts that can potentially contribute to the target production even when there are no sets of knockouts that have the target production in the initial population.

### 3.2 Improvement of target production yield by using FastPros and OptKnock

Although FastPros provides sets of reaction knockout candidates as described earlier, these knockout sets did not always result in the desired high production yield of target metabolites required for engineering metabolic design. The sign of 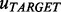 represents only whether the production of a target metabolite is beneficial for growth and the absolute value of this score does not correspond to its productivity. Therefore, iterative screening using 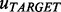 is insufficient for identifying the reaction knockouts that cause high-target productivity.

**Fig. 2.**
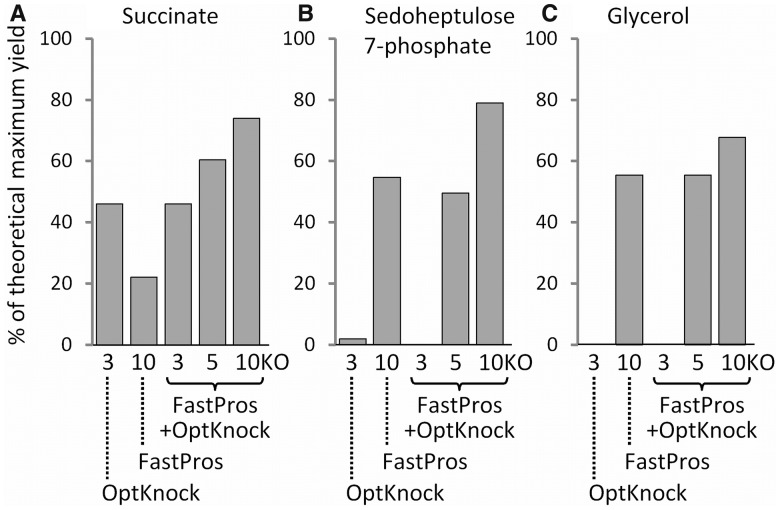
Estimated production yields of (**A**) succinate, (**B**) sedoheptulose 7-phosphate and (**C**) glycerol by OptKnock, FastPros and FastPros-based OptKnock. Maximum knockout number was set to 3 in OptKnock, 10 in FastPros and 3, 5 and 10 in FastPros-based OptKnock

Therefore, we proposed an alternative algorithm for knockout screening for high-target production using both FastPros and OptKnock. As mentioned earlier in the text, iterative screening using 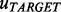 generates sets of reaction knockouts that increase the score to a positive value. It was anticipated that these screened reaction knockouts have a relatively large impact on the metabolic fluxes changing toward production of the target. Accordingly, to obtain a set of reaction knockouts resulting in an optimal yield of a target metabolite, it is appropriate to search for an optimal combination of knockouts from those screened based on 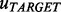 values. To obtain such a combination, we used the OptKnock algorithm, in which candidates of reaction knockouts were restricted to those obtained in the FastPros screening. Various sets of knockouts resulting in positive or zero 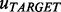 values were collected, of which 30 unique reaction knockouts that contributed to the highest production yields were used as knockout candidates for the OptKnock screening with several maximum numbers of knockouts (3, 5 and 10 KO). In this analysis, we considered the production of 380 target metabolites whose productions were predicted by adding no more than 10 reaction knockouts by FastPros (Supplementary Table S2). We found that for 106 (28%) of the target metabolites, production yields increased by >5% of the theoretical maximum yield (TMY) in comparison with the cases in which FastPros only was used. For example, the production yields of succinate and sedoheptulose 7-phosphate were predicted by FastPros only to be 22 and 55% of the TMY, respectively, but were 74 and 79% of TMY when using FastPros-based OptKnock screening (Figure 2A and B). In another example, the production yield of glycerol was estimated to be 68% of the TMY by FastPros-based OptKnock screening, but zero using OptKnock only (Fig. 2C). These results indicate that the combination of FastPros and OptKnock can provide appropriate sets of reaction knockouts for high-target production yields. Moreover, the target production yields using both FastPros and OptKnock compared favorably with those using OptGene or GDLS (Supplementary Fig. S3), indicating the proposed approach can be an alternative to the other knockout screening methods.

### 3.3 Examples of knockout screening by FastPros

**Fig. 3.**
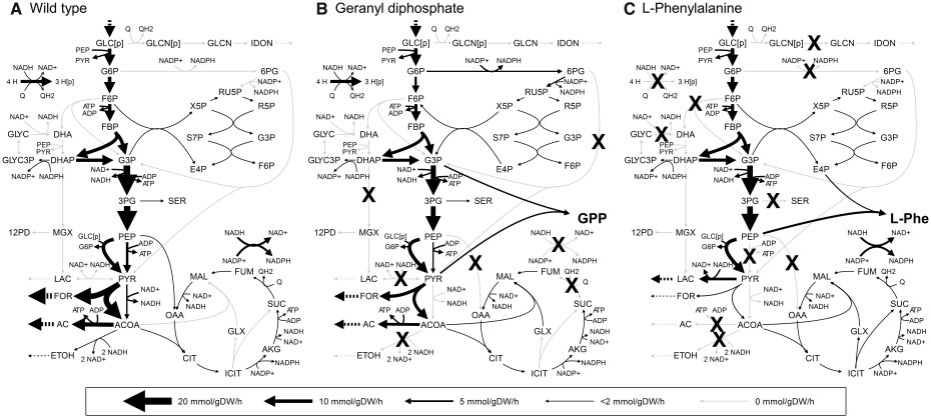
Metabolic flux profiles of (**A**) wild-type strain, (**B**) GPP production strain and (**C**) l-phenylalanine production strain. Arrow width represents metabolic flux of the reaction. Solid arrows and dashed arrows indicate reactions in cytosol and exchange reactions, respectively. Crosses represent reaction knockouts screened by our method. Abbreviations are described in Supplementary Material

Geranyl diphosphate (GPP) can be biologically converted into geraniol, which is an aromatic material commonly used in perfume. GPP is also an intermediate in a terpenoid biosynthesis pathway (the non-mevalonate pathway). Accordingly, reaction knockouts that increase the biosynthesis flux toward GPP can increase the production of terpenoids in *E.coli*. Therefore, the design of appropriate metabolic networks for GPP production is valuable for engineering applications. We performed FastPros screening for GPP production and identified 88 sets of reaction knockouts that resulted in the production of GPP when biomass production was maximized. These knockout sets contained 25 unique reactions that were selected for the OptKnock screening (Supplementary Table S3). Knockouts of seven reactions resulted in the highest yield of GPP, 0.34 g/g-glucose (53.3% of TMY). Figure 3A and B shows the estimated flux profiles of wild type and of the identified knockouts, respectively. The production of GPP from glyceraldehyde 3-phosphate and pyruvate as precursors requires the reducing power of NADH and NADPH. Therefore, competing NADH and NADPH oxidization pathways, such as ethanol and lactate production pathways, were mainly disrupted for GPP production (see details in Supplementary Material).

l-Phenylalanine (l-Phe) is an aromatic amino acid with many applications in the food and pharmaceutical industries, such as aspartame. Because chemical synthesis of l-Phe generates racemic mixtures of d- and l-Phe, the production of pure l-Phe in bioprocesses is important. *E.coli* contains a natural l-Phe biosynthetic pathway that has been used in commercial production. We performed FastPros screening and identified 57 knockout sets for the production of l-Phe, including 44 unique reactions. Of these, 30 reactions contributing higher production yields were selected for OptKnock screening (Supplementary Table S4). A production yield of 0.27 g/g-glucose (58.0% of TMY) was estimated for the knockout of 10 reactions. Figure 3C shows the estimated flux profile of the obtained l-Phe production network. One mole of l-Phe is synthesized from 2 mol of phosphoenolpyruvate (PEP) in glycolysis and 1 mol of erythrose 4-phosphate (E4P) in the pentose phosphate pathway. Accordingly, reactions that convert PEP or other metabolites in upper glycolysis into pyruvate were knocked out, enhancing the carbon flow from PEP and E4P to l-Phe (see details in Supplementary Material).

### 3.4 Similarity of knocked out reactions

The results of FastPros screening demonstrated that metabolites often share the same reaction knockouts for their production. Here, we analyzed the similarity of knockout candidate sets among the target metabolites, with the aim of identifying a common knockout set for the production of a specific class of metabolites. The identification of such common knockout sets enables the design of a common parent strain, from which production strains for various target metabolites can be generated by adding a variety of additional reaction knockouts to the parent strain.

**Fig. 4.**
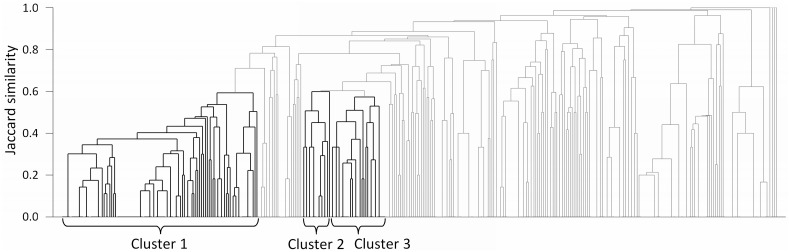
Dendrogram of the target metabolites based on screened reaction knockouts for their production. Metabolites in the clusters generally belong to same metabolic class. For example, majorities of metabolites in Clusters 1, 2 and 3 are lipid and their derivatives, aromatic amino acids and precursors and sugars and sugar phosphates, respectively. Metabolites in the same cluster share the same reaction knockouts for their production (see details in Supplementary Table S5)

Figure 4 illustrates a dendrogram of the production targets, in which the similarity between two targets has been calculated by the membership-based Jaccard similarity coefficient (Levanodowsky and Winter, 1971) of the reaction knockouts sets obtained by FastPros-based OptKnock screening. It can be seen that several clusters of metabolites exist, and we found that metabolites belonging to a same cluster tend to be close in the metabolic network (see Supplementary Table S5). For example, Cluster 1 in Figure 4 consisted of 103 metabolites, most of which were lipids or their derivatives such as decanoate, phosphatidylglycerol and GPP. In this cluster, knockouts of the alcohol dehydrogenase (ADH), lactate dehydrogenase (LDH), methylglyoxal synthase (MGS), and PEP carboxylase (PPC) reactions were shared among >90% of these targets, suggesting these knockouts could be a basic strategy for the production of metabolites (i.e. lipids and their derivatives) in this cluster. Cluster 2 comprised 15 target metabolites, which consisted of intermediates and derivatives of the aromatic acid biosynthesis pathway (3-dehydro-shikimate, l-Phe, etc.), and knockouts of PPC, pyruvate carboxylase, MGS and phosphoglycerate dehydrogenase were shared by the metabolites in this cluster. Cluster 3 consisted of 29 target metabolites, most of which were sugars or sugar phosphates, the production of which shared knockouts of PPC, pyruvate kinase, phosphoglycerate dehydrogenase and glucose 6-phosphate dehydrogenase. The detailed mechanisms to produce the metabolites in Clusters 1, 2 and 3 are discussed in the Supplementary Material.

## 4 DISCUSSION

We have developed a novel algorithm, FastPros, to screen sets of reaction knockouts that produce target metabolites. We used a screening score, 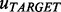, which is calculated as a shadow price in a linear programming problem, to evaluate the potential of target production under the condition of biomass production maximization. It should be stressed that FastPros enables us to identify reaction knockout sets with a large number of reactions, which has hitherto been difficult to achieve by both comprehensive and iterative screening methods. We confirmed that FastPros has significant advantages for screening knockout sets to the widely used methods OptGene and GDLS (Supplementary Fig. S2). We expect that the use of FastPros will greatly accelerate computer-aided metabolic design for industrial bioproductions.

The FastPros screening method we have proposed can be used in collaboration with other *in silico* methods for metabolic engineering, for example, for the production of non-native metabolites. Although in this study we considered only the screening of reaction knockouts from a wild-type metabolic network, FastPros screening would be applicable, for example, after the addition of heterologous reaction pathways to produce desired non-native targets (Chatsurachai *et al.*, 2012; Cho *et al.*, 2010; Pharkya *et al.*, 2004). The combinatorial use of FastPros and other network-expansion algorithms enables us to design appropriate metabolic networks for non-native metabolite productions. As another example, metabolic networks designed by FastPros screening can be used as initial conditions for other network-design algorithms, such as OptKnock and OptStrain (Pharkya *et al.*, 2004). Knockout sets leading to higher target productivities can effectively prune suboptimal solutions, thereby reducing computation time to identify the global optimal solution in comprehensive screenings (Kim *et al.*, 2011). Moreover, some previous reports have successfully incorporated the effect of gene upregulations for target metabolite productions (Kim and Reed, 2010; Pharkya and Maranas, 2006). This strategy for the evaluation of gene upregulation can be integrated into FastPros analysis, which enables us to screen sets of gene manipulations, including both gene deletions and upregulations, to realize target metabolite prediction with high productivity.

Although FastPros is based on reaction knockouts, we also considered gene deletions. As mentioned in Section 2, reactions encoded by the same gene sets in a reduced model are knocked out simultaneously (e.g. two transketolase reactions are knocked out at once, as they both are encoded by the same *tktA* and *tktB* gene sets), which avoids cases where only a part of the reactions encoded by the same gene sets is disrupted *in silico*. By considering the relationship between metabolic reactions and genes encoding corresponding enzymes, we can develop strategies for gene manipulation using *in silico* screening by FastPros.

The algorithm of FastPros is based on the assumption of biomass production maximization, that is, that metabolic fluxes are organized to achieve an optimal profile for cellular growth. Of course, this assumption is not always the case experimentally. If a strain after the predicted reaction knockouts cannot be obtained due to no or slow growth, screening should be performed again with consideration of lethal or growth-defect knockouts. The integration of data on synthetic lethality (Typas *et al.*, 2008) to FastPros screening might facilitate avoiding mismatches between predicted growth and experimental results. Setting larger growth thresholds in FastPros can also help avoid mutant strains that have growth defects. An alternative way to avoid growth defects is to use an appropriate selection score, which ensures the cell growth. For example, the Biomass-Product Coupled Yield is calculated as a product of production yield and cell growth yield (Patil *et al.*, 2005). Using Biomass-Product Coupled Yield as the selection score in the FastPros analysis, we can expect to obtain knockout sets that result in both the target production and active growth. Even if a strain after the predicted reaction knockouts is successfully constructed, it can remain in a non-optimal metabolic state. Conflicts between experiments and *in silico* predictions can be eliminated by experimental evolution. Several studies have demonstrated that when a microorganism strain is cultured for an extended period to select individuals with optimized growth under a given condition, the realized flux profile gradually approaches the calculated optimal flux profile (Fong *et al.*, 2005; Lewis *et al.*, 2010). The combination of *in silico* screening and experimental evolution that achieves an optimal flux profile experimentally is a potentially practical application for obtaining valuable strains for bioproduction.

In conclusion, we have developed a novel computational algorithm, FastPros, that accelerates microbial strain improvement and have demonstrated the applicability of FastPros to cell-wide metabolite productions in *E.coli*. 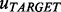 was adopted as the screening score to identify those reaction knockouts that have greater potential for target metabolite production. This score could be readily calculated as a shadow price in modified FBA simulations. We identified numerous metabolites in *E.coli* whose production was enhanced under biomass production maximization only when multiple reactions were simultaneously knocked out. We expect that our algorithm and the concept of 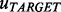 will improve metabolic engineering technologies and facilitate biological production of a broad range of valuable chemicals.

## Supplementary Material

Supplementary Data
