# Supplementary material for: FastPros: screening of reaction knockout strategies for metabolic engineering
Source: Bioinformatics. 2013 Nov 19;30(7):981–7. doi: 10.1093/bioinformatics/btt672 (PMC3967105; doi:10.1093/bioinformatics/btt672)
Supplement: Supplementary Data [file supp_30_7_981__index.html]

FastPros: screening of reaction knockout strategies for metabolic engineering — Supplementary Data 

# FastPros: screening of reaction knockout strategies for metabolic engineering

## Supplementary Data

files

**Files in this Data Supplement:**

- Supplementary Data - docx file
