# Supplementary material for: FastPros: screening of reaction knockout strategies for metabolic engineering
Source: Bioinformatics. 2013 Nov 19;30(7):981–7. doi: 10.1093/bioinformatics/btt672 (PMC3967105; doi:10.1093/bioinformatics/btt672)
Supplement: Supplementary Data [file supp_btt672_supplementary_data_revised.docx]

**Supplementary Method**

**Screening by OptGene and GDLS**

OptGene and GDLS were performed, as previously described (Lun *et al.*, 2009; Patil *et al.*, 2005). As in the FastPros screening, reduced metabolic models were used and the maximum numbers of knockouts were set to 25. In GDLS, the number of search paths and neighborhood size were set to 2 and 2, respectively.

OptKnock is known to work poorly with the glpk MILP solver (described in the testOptKnock script in COBRA toolbox (Schellenberger *et al.*, 2011)). We found that in the cases of 5 metabolites out of total 625 metabolites, the obtained production fluxes by OptKnock (3 knockouts) with GLPK are smaller than those obtained by comprehensive calculation in which all possible triple knockouts were calculated. The metabolites of such mismatches are presented in Supplementary Table S6.

**Supplementary Texts**

**1: Abbreviations in Fig. 3**

12PD, 1,2-propanediol; 6PG, 6-phospho-gluconate; AC, acetate; ACOA, acetyl-CoA; CIT, citrate; DHA, dihydroxyacetone; DHAP, dihydroxyacetone phosphate; E4P, erythrose 4-phosphate; ETOH, ethanol; F6P, fructose 6-phosphate; FBP, fructose 1,6-bisphosphate; FOR, formate; FUM, fumarate; G3P, glyceraldehyde 3-phosphate; G6P, glucose 6-phosphate; GLC, glucose; GLC[p], glucose (periplasm); GLCN, gluconate; GLCN[p], gluconate (periplasm); GLX, glyoxylate; GLYC, glycerol; GPP, Gerany diphosphate; GLYC3P, glycerol 3-phosphate; ICIT, isocitrate; IDON, idonate; LAC, d-lactate; L-Phe, l-Phenylalanine; MAL, malate; MGX, methylglyoxal; OAA, oxaloacetate; PEP, phosphoenolpyruvate; PYR, pyruvate; Q, ubiquinone; QH2, ubiquinol; R5P, ribose 5-phosphate; RU5P, ribulose 5-phosphate; S7P, sedoheptulose 7-phosphate; SER, l-Serine; SUC, succinate; X5P, xylulose 5-phosphate.

**2: Mechanisms to produce geranyl diphosphate (GPP) in Fig. 3**

Knockouts of seven reactions, namely, those catalyzed by alcohol dehydrogenase (ADH), lactate dehydrogenase (LDH), phosphoenolpyruvate carboxylase (PPC), methylglyoxal synthase (MGS), transhydrogenase (THD), 6-phosphogluconate dehydratase (EDD) and succinate dehydrogenase (SDH) resulted in the highest yield of GPP, 0.34 g/g-glucose (53.3% of TMY). For the production of 1 mol GPP, 2 mol of glyceraldehyde 3-phosphate (G3P) and 2 mol of pyruvate are used as precursors, and 4 mol of NADH and 2 mol of NADPH are oxidized. For the production of GPP, therefore, competing NADH and NADPH oxidization pathways, including the ethanol, lactate and succinate production pathways, were disrupted by the knockout of ADH, LDH, and PPC. Knockout of MGS disrupted the production of 1,2-propanediol, which also requires NADH oxidization. Knockout of THD inhibited the electron transfer from NADH to NADP^+^, avoiding the L-valine production accompanied with NADPH consumption. Finally, knockout of EDD disrupted excess carbon flow to lower glycolysis via the Entner-Doudroff (ED) pathway, which enhanced the G3P supply for GPP production. The above reaction knockouts contributed the bulk of the GPP yield (0.31 g/g-glucose). SDH disruption caused a slight decrease of the cell growth rate, and surplus carbon was shunted toward GPP biosynthesis.

**3: Mechanisms to produce L-phenylalanine (L-Phe) in Fig. 3**

A production yield of 0.27 g/g-glucose (58.0% of TMY) was estimated for the knockout of the following 10 reactions: pyruvate kinase (PYK), fructose 6-phosphate aldolase (F6PA), PPC, glucose 6-phosphate dehydrogenase (G6PDH), phosphoglycerate dehydrogenase (PGCD), glycerol dehydrogenase (GLYCD), phosphotransacetylase (PTA), ADH, NADH dehydrogenase (NDH) and gluconate transporter (GLCNt). One mole of L-Phe is synthesized from 2 mol of phosphoenol- pyruvate (PEP) in glycolysis and 1 mol of erythrose 4-phosphate in the pentose phosphate pathway with oxidization of 2 mol of NADPH. Accordingly, knockouts of the PYK, F6PA, and PPC-catalyzed reactions were selected since they decreased conversion flux from PEP to pyruvate, thereby favoring the conversion of PEP to L-Phe. Knockout of G6PDH disrupted the carbon flux of the ED pathway, followed by pyruvate biosynthesis. Knockout of PGCD disrupted the conversion of 3-phosphoglycerate to L-serine and subsequently to pyruvate in a reaction catalyzed by serine deaminase. In this strain, the L-serine required for cell growth was synthesized from glycine, which was itself synthesized by l-threonine degradation. Knockout of GLYCD disrupted the conversion of PEP to pyruvate via the dihydroxyacetone phosphotransferase-catalyzed reaction. Deletion of PTA disrupted acetate production with ATP generation, with additional generation of ATP from phosphoglycerate kinase in glycolysis, increasing the PEP supply. ADH disruption inhibited NADH oxidization during ethanol biosynthesis, followed by activation of the THD reaction to oxidize NADH, with reduction of NADP^+^. Together, these reaction knockouts contributed to almost all the L-Phe yield (0.26 g/g-glucose). Additional disruptions of both NDH and GLCNt decreased the biomass production to increase L-Phe biosynthesis. Only NDH disruption without CLCNt knockout resulted in idonate biosynthesis through GLCNt without enhancing L-Phe production.

**4: Mechanisms to produce metabolites in Clusters 1, 2 & 3 in Fig. 4**

Cluster 1 consisted of 103 metabolites, most of which were lipids or their derivatives such as decanoate, phosphatidylglycerol and geranyl diphosphate. In this cluster, knockouts of alcohol dehydrogenase, lactate dehydrogenase, methylglyoxal synthase (MGS), and PEP carboxylase (PPC) reactions were shared among the more than 90% of these targets. Carbon chain elongation in lipid biosynthesis requires the reducing power of NADPH and NADH and, consequently, the knockouts of the above reactions associated with oxidation of NADH or NADPH were screened to enhance lipid biosynthesis.

Cluster 2 comprised 15 target metabolites, which consisted of intermediates and derivatives of the aromatic acid biosynthesis pathway (3-dehydroshikimate, L-Phe, 4-hydroxybenzoate, etc.), and knockouts of PPC, pyruvate carboxylase (PYK), MGS, and phsphoglycerate dehydrogenase (PGCD) were shared by more than 90% of the metabolites in this cluster. PEP is an important starting metabolite for aromatic acid biosynthesis, and disruptions of PPC, PYK, MGS and PGCD (which inhibits the conversion of PEP into pyruvate) can therefore activate the PEP supply for aromatic acid biosynthesis.

Cluster 3 consisted of 29 target metabolites, most of which were sugars or sugar phosphates, the production of which share knockouts of PPC, PYK, PGCD and glucose 6-phosphate dehydrogenase. These reaction knockouts inactivated pyruvate biosynthesis from intermediates in the glycolysis or pentose phosphate pathway and enhanced sugar or sugar phosphate biosynthesis from the upper stream of the glycolysis.

**Figure. S1. Schematic representation of selection procedure in FastPros.**


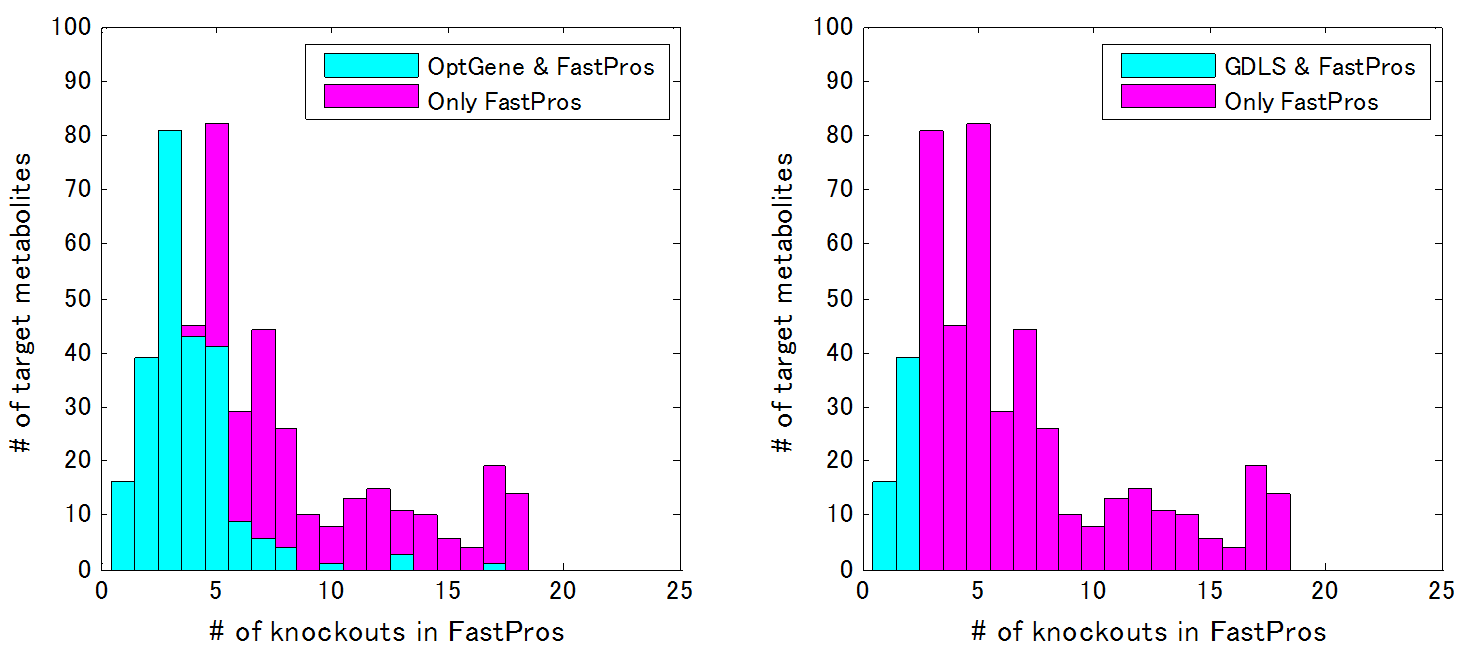


**Figure. S2. Distribution of the minimum number of reaction knockouts necessary for target metabolite production.** (left) Identified by FastPros only and by both OptGene and FastPros. (right) Identified by FastPros only and by both GDLS and FastPros. Among the 247 metabolites screened by OptGene, 244 were also screened by FastPros; of the 55 metabolites screened by GDLS, all were screened by FastPros. The parameters for OptGene were as follows: population size, 125; maximum number of generations, 5000; maximum number of knockouts, 25; and mutation rate, 1/(# of allowable knockout sets). The parameters for GDLS were: search path, 2; neighborhood size, 2; and maximum number of knockouts, 25. These parameters were obtained from the original reports (Lun *et al*., 2009; Patil *et al*., 2005). In these conditions, the average calculation time to obtain knockout sets for one target metabolite (up to 25 knockouts) was 6.2, 5.8, 0.1 hours for FastPros, OptGene, and GDLS, respectively. See Supplementary Method for details of OptGene and GDLS screenings.

**
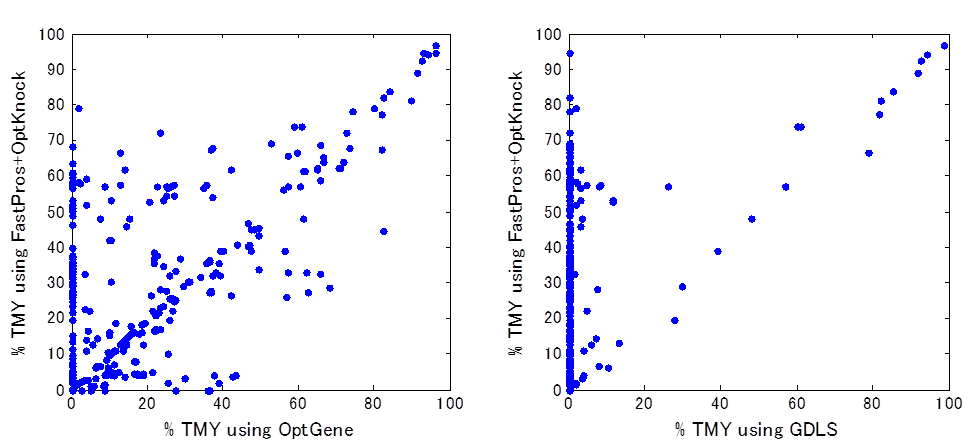
**

**Figure. S3. Predicted production yields by (left) OptGene vs FastPros+OptKnock, and (right) GDLS vs FastPros+OptKnock.** Each point represents the predicted production yield of each target metabolite. Yields are shown in % of the theoretical maximum yield (TMY).

**Table S3. List of the reactions allowed to be knocked out for geranyl diphophate production in FastPros-based OptKnock**

| Reactions allowed to be knocked out |
| --- |
| EX_etoh(e)/ETOHtex/ALCD2x/ETOHt2rpp,ACALD |
| LDH_D |
| MDH |
| MGSA |
| THD2pp |
| EDA/EDD |
| EX_lac_D(e)/D_LACtex/D_LACt2pp |
| GLYCDx,LALDO2x/LCARR |
| ALR2/ALR4x |
| PPC |
| GLUDy |
| EX_acald(e)/ACALDtex/ACALDtpp,H2St1pp/EX_h2s(e)/H2Stex |
| ILEt2rpp,LEUt2rpp,VALt2rpp |
| NADH16pp,NADH17pp,NADH18pp |
| FUM |
| GLCt2pp |
| FRD2,FRD3 |
| SUCDi |
| ATPS4rpp |
| ICL |
| ASPT |
| FBA |
| PFK |
| PGL/G6PDH2r |
| PGCD/PSERT/PSP_L |

Reaction names are abbreviation defined in the metabolic model iAF1260 (Feist *et al.*, 2007). “reaction A/reaction B”, set of merged reactions without branching; “reaction C,reaction D”, set of reactions encoded by the same gene sets.

**Table S4. List of the reactions allowed to be knocked out for l-phenylalanine production in FastPros-based OptKnock**

| Reactions allowed to be knocked out |
| --- |
| PYK |
| F6PA |
| PPC |
| PGL/G6PDH2r |
| PGCD/PSERT/PSP_L |
| GLYCDx,LALDO2x/LCARR |
| ACKr/PTAr |
| EX_etoh(e)/ETOHtex/ALCD2x/ETOHt2rpp,ACALD |
| LDH_D |
| MGSA |
| PDH |
| EDA/EDD |
| DHAPT |
| GLCptspp |
| TPI |
| F6PP,G3PT,G6PP,MN6PP,R5PP |
| MDH |
| GND |
| TALA |
| GNK |
| GLCNt2rpp |
| GLCDpp |
| FRD2,FRD3 |
| PGI |
| HEX1 |
| AKGDH |
| NADH16pp,NADH17pp,NADH18pp |
| GHMT2r |
| MTHFC/MTHFD |
| GLYAT |

Reaction names are abbreviations defined in the metabolic model iAF1260 (Feist *et al.*, 2007). “reaction A/reaction B”, set of merged reactions without branching; “reaction C,reaction D”, set of reactions encoded by the same gene sets.

**Table S5. List of the target metabolites included in Clusters 1, 2 and 3 in Fig. 4**

| Cluster | Target metabolites (alphabetical order) | | | |
| --- | --- | --- | --- | --- |
| Cluster 1 | 12dgr120 | 12dgr140 | 12dgr141 | 12dgr160 |
|  | 12dgr161 | 12dgr180 | 12dgr181 | 1ddecg3p |
|  | 1hdec9eg3p | 1hdecg3p | 1odec11eg3p | 1odecg3p |
|  | 1tdec7eg3p | 2agpe160 | 2agpe180 | 2agpe181 |
|  | 2agpg120 | 2agpg140 | 2agpg141 | 2agpg160 |
|  | 2agpg161 | 2agpg180 | 2agpg181 | 2ddecg3p |
|  | 2dmmq8 | 2dmmql8 | 2hdec9eg3p | 2hdecg3p |
|  | 2odec11eg3p | 2odecg3p | 2ohph | 2tdec7eg3p |
|  | 2tdecg3p | 3ophb | apg120 | apg140 |
|  | apg141 | apg160 | apg161 | apg180 |
|  | apg181 | cdpdhdecg | cdpdodecg | cdpdtdecg |
|  | dca | ddca | dmpp | frdp |
|  | grdp | hdca | hdcea | ipdp |
|  | kdo2lipid4L | kdo2lipid4p | kdolipid4 | lipa |
|  | lipa_cold | lipidA | lipidAds | lipidX |
|  | mql8 | ocdca | ocdcea | octa |
|  | octdp | pa140 | pa141 | pa160 |
|  | pa161 | pa180 | pa181 | pe120 |
|  | pe140 | pe141 | pe160 | pe161 |
|  | pe181 | pg120 | pg140 | pg141 |
|  | pg161 | pg180 | pg181 | pgp120 |
|  | pgp140 | pgp141 | pgp161 | pgp181 |
|  | ps120 | ps140 | ps141 | ps160 |
|  | ps161 | ps180 | ps181 | q8 |
|  | ttdca | ttdcea | uLa4fn | udcpdp |
|  | udcpp | unagamu | unagamuf |  |
| Cluster 2 | 23ddhb | 23dhb | 3dhq | 3dhsk |
|  | 4hbz | fdp | g3p | gam1p |
|  | gam6p | gmhep17bp | indole | phe_L |
|  | ru5p_L | tagdp_D | xu5p_D |  |

(Table S5 continued)

| Cluster 3 | 2dda7p | 2ddg6p | 6pgc | acglc_D |
| --- | --- | --- | --- | --- |
|  | all6p | allul6p | ara5p | bglycogen |
|  | f6p | fru | g1p | g6p |
|  | gal1p | glycogen | gmhep1p | kdo8p |
|  | kdo | man1p | man6p | man |
|  | mnl1p | r1p | r5p | rib_D |
|  | ru5p_D | sbt6p | skm | tre6p |
|  | tre |  |  |  |

Metabolite names are abbreviations defined in the metabolic model iAF1260 (Feist *et al.*, 2007).

**Table S6. List of incorrect solutions of OptKnock (3KO).**

| Target metabolites  (Abbr.) | Predicted production yields by OptKnock  [% of TMY] | Predicted production yields by a comprehensive screening method(Ohno *et al.*, 2013) [% of TMY] | Difference in the production yields between the two screening methods  [% of TMY] |
| --- | --- | --- | --- |
| 2dhp | 15.7 | 76.2 | 60.4 |
| cmp | 0 | 1.2 | 1.2 |
| gggagicolipa | 0 | 16.2 | 16.2 |
| nmn | 0 | 8.2 | 8.2 |
| thmpp | 0 | 13.9 | 13.9 |

Abbreviations: 2dhp, 2-dehydropantoate; cmp, CMP; gggagicolipa, glucosyl-glucosyl-galactosyl- glucosyl-inner core oligosaccharide lipid A; nmn, NMN; thmpp, Thiamine diphosphate; TMY, theoretical maximum yield.

**References**

Feist,A.M. *et al.* (2007) A genome-scale metabolic reconstruction for *Escherichia coli* K-12 MG1655 that accounts for 1260 ORFs and thermodynamic information. *Mol. Syst. Biol.*, **3**, 121.

Lun,D.S. *et al.* (2009) Large-scale identification of genetic design strategies using local search. *Mol. Syst. Biol.*, **5**, 296.

Ohno,S. *et al.* (2013) In silico screening of triple reaction knockout *Escherichia coli* strains for overproduction of useful metabolites. *J. Biosci. Bioeng.*, **115**, 221–8.

Patil,K.R. *et al.* (2005) Evolutionary programming as a platform for in silico metabolic engineering. *BMC Bioinformatics*, **6**, 308.

Schellenberger,J. *et al.* (2011) Quantitative prediction of cellular metabolism with constraint-based models: the COBRA Toolbox v2.0. *Nat. Protoc.*, **6**, 1290–307.
